# Non-invasive obstetric anal sphincter injury diagnostics using impedance spectroscopy

**DOI:** 10.1038/s41598-019-43637-1

**Published:** 2019-05-08

**Authors:** Katarzyna Borycka-Kiciak, Marcel Młyńczak, Adam Kiciak, Piotr Pietrzak, Adam Dziki

**Affiliations:** 1 Department of General, Oncological and Gastrointestinal Surgery, Orlowski Hospital, Centre of Postgraduate Medical Education, Warsaw, Poland; 2 Warsaw University of Technology, Faculty of Mechatronics, Institute of Metrology and Biomedical, Warsaw, Poland; 3 Department of Physiological Sciences, Faculty of Veterinary Medicine, Warsaw University of Life Sciences, Warsaw, Poland; 4 Department of General and Colorectal Surgery, Medical University of Łódź, Łódź, Poland

**Keywords:** Electrodiagnosis, Trauma, Anal diseases

## Abstract

Obstetric anal sphincter injuries are the most common cause of fecal incontinence in women yet remain under-diagnosed. The aim of this study was to assess the suitability of impedance spectroscopy for diagnosing sphincter injuries arising during delivery. This was a prospective single-center study. 22 female patients were included: 10 with symptoms of sphincter dysfunction, in the early postpartum period, and 12 unaffected, in the distant period of more than 2 years after natural delivery. The presence, extent and severity of anal sphincters injury was assessed by measuring the sphincter parameters in physical examination, the degree of sphincter damage in endoanal ultrasound imaging and the sphincters function parameters in anorectal manometry. All measurements were used as references and compared with the outcomes from the impedance spectroscopy models. Impedance spectroscopy showed the highest precision (with mean accuracy of 83.9%) in relation to transanal ultrasonography. 74.1% of its results corresponded to the results of rectal physical examination and 76.7% - to those of anorectal manometry. The method showed the highest accuracy in the assessment of the sphincter’s parameters, both anatomically and functionally. New impedance spectroscopy techniques hold promise for detecting obstetric anal sphincter injuries.

## Introduction

Obstetric perineal tears associated with anal sphincter injury (third- and fourth-degree) are the most common cause of fecal incontinence in women^1^. The sphincter defect may be further exacerbated by direct compression or stretching of the pudendal nerve, as neurogenic damage, not detectable through imaging. The set of incontinence symptoms (flatal incontinence, passive soiling, incontinence of liquid or solid stool), having a very destructive impact on quality of life^2,3^, may appear soon after delivery or, more often, many years later^4–6^. Studies have shown that 10–30% of women with obstetric anal sphincter injuries (OASI) will develop symptoms of fecal incontinence later in life^5,6^. The reported incidence of OASI varies between 3% and 20% of deliveries^4,7–10^ and even this is probably underestimated. Ultrasonographic evidence of OASI has been found in 27–35% of women after first vaginal delivery and 3–12% after subsequent ones^11,12^. Unfortunately, over 80% of these injuries remain unrecognized in the delivery suite^12,13^.

Although there is a wide range of tools (including ultrasound imaging, anorectal manometry and nerve conduction studies) intended for diagnosing OASI, their availability, especially in the early postpartum period, is limited. The additional weakness of most of these tests is a lack of correlation between functional or morphological findings and clinical scoring of the disease^14^.

Endoanal ultrasonography, as the gold standard in the diagnosis of OASI, reveals structural changes of the internal or external sphincter muscles (detecting the anatomic causes of fecal incontinence) whereas anorectal manometry collects data about the anal canal pressures and the rectoanal inhibitory reflex being helpful in the diagnosis of functional disorders. Both tests, however, are usually delayed until a few weeks after the injury and require highly skilled interpretation^15^. Physical rectal examination has thus far remained the most appropriate diagnostic tool in early detection of OASI^16,17^. However, its effectiveness also depends on the medical staff’s experience and it cannot be used as a proper test.

Therefore, despite the role that the latest guidelines assign to ultrasound imaging^17,18^, there is still a great clinical need for a simple, useful tool for early diagnosis of anal sphincter damage^13^.

One of the most promising tools widely used in the diagnosis of different pathologies is impedance spectroscopy. Electrical impedance has been identified as a physical marker of the condition of tissues, varying with their microscopic structure, hydration, electrolyte concentrations and other parameters^19,20^. Therefore, its value seems to reflect simultaneously the anatomical and functional statuses of the evaluated tissues.

Thanks to these physical properties, the impedance may be useful for detecting sphincter damage^21–24^, especially in the postpartum diagnosis of hidden, otherwise-unrecognizable injuries.

The objective of the project was to assess the suitability of impedance spectroscopy as a tool for detection of obstetric anal sphincter injuries and can be used within the first days after injury, even by inexperienced doctors.

## Materials and Methods

The study protocol was defined in accordance with the GCP guidelines and approved by the Ethics Committee of the Medical Chamber in Warsaw (approval no. KB/977/15). Patients gave written informed consent before entering the study.

### The study groups

The study population comprised 22 subjects, all female, aged 36.3 ± 9.8 (all ages expressed as mean ± standard deviation), recruited from among patients visiting the NASMED Medical Center in Warsaw between December 2017 and February 2018. 10 patients (as the group A), being 6–8 weeks after vaginal delivery, with symptoms of sphincter dysfunction, were selected in a consecutive manner from among patients in the obstetrics outpatient clinic. The group B consisted of 12 women, after delivery in the past, selected from patients of the surgical outpatient clinic, treated for proctological reasons but without any symptoms of sphincter dysfunction. The entire study population was checked for the presence of other risk factors for fecal incontinence, like neurological disease, diabetes mellitus, previous anorectal surgery, urinary incontinence, obesity and history of smoking – no other risk factors were found.

The women in group A, aged 30.9 ± 3.7 years, were primiparous (9/10) or giving birth for the second time (1/10), and had reported flatal incontinence, liquid seepage and/or liquid incontinence. The incontinence rates were assessed to have a mean Wexner score of 4.5 (2–8). In all cases, the second phase of delivery had been prolonged; in 4 cases, oxytocin had been used. Episiotomy had been carried out in all 10 women. In 2 cases, the delivery was vacuum-assisted, and in 2 cases, forceps-assisted. The mean neonatal body weight was 3530 g (in the range of 2670–4000 g). In 6 cases, only second-degree perineal tears had been diagnosed at delivery; in the other 4, OASIs had been confirmed (1 fourth- and 3 third-degree perineal tears) and primary sphincter repair performed.

The women in group B, aged 40.8 ± 11.0 years, had given birth more than 2 years ago (7/12 were primiparous and 5/12 multiparous), and did not report any symptoms of fecal incontinence (Wexner score of 0). The primary reasons for the surgical visit were third-degree hemorrhoidal disease (9/12) and ulcerative colitis (3/12) currently in remission.

### Study design

The duration of the study for each patient was 4 weeks. In all cases, after obtaining informed consent (V0), the physical rectal examination was performed during visit 1 (V1). The severity of fecal incontinence symptoms was assessed according to the Wexner incontinence scale. Subsequently, impedance spectroscopy assessment was performed. The measurements were raw physical parameters, which do not need any medical interpretation. The detailed description of the method and the measurement technique is presented in the next subsection. The examination was short and painless, each time performed with the subject lying on her left side with their sphincter muscles relaxed, and taking no longer than 1 minute. The patients did not report any additional discomfort associated with the impedance spectroscopy measurement.

Over the 3 following weeks, patients underwent standard diagnostics tests used to detect pelvic floor muscle injuries: endoanal ultrasonography (using a BK Medical Colorectal ultrasound scanner type Flex Focus 1202 with anorectal transducer type 2052- from BK Medical A/S, Mileparken 34, DK-2730 Herlev, Denmark), and anorectal manometry (a CLIPER High Resolution Manometry System using a 12-channel micro-relay catheter with a diameter of 4.2 mm, with 10 of the sensors spaced at 6 mm intervals and 2 rectal sensors at the end). During the control visit (V2), the test results were evaluated independently from impedance spectroscopy data, and final diagnosis was made. Then, it was treated as an output for the model connecting impedance data with clinical results.

### Impedance spectroscopy measurements

Impedance spectroscopy is one of the techniques, which uses the measurement of the electrical properties (impedance parameters) of the tissues. The impedance should be measured indirectly. In the most commonly used configuration, a setting involving 4 electrodes is used, in which the first pair delivers an “application” current (usually sinusoidal, with constant amplitude, independent of the measured impedance), while the second pair measures voltage changes, which are strictly related to the impedance by Ohm’s law.

Impedance techniques are widely used in various fields of medicine. For example, impedance cardiography is intended to estimate stroke volume indirectly^25^. On the other hand, impedance pneumography is utilized to measure tidal volume and air-flow without the need to use a special face mask or mouthpiece^26–28^. Bioelectrical impedance analysis (BIA) is commonly used for estimating body composition^29^. Finally, electrical impedance tomography (EIT) – imaging method using impedance – was preliminarily utilized for prostate cancer detection^30^.

From impedance, which originally consists of resistance and reactance, one can calculate the impedance modulus and the phase shift between the voltage and the current, and present them with respect to current frequency. This is the main point of the spectroscopy, which provides information about the electrical properties of tissues at their different depths, over a wide frequency spectrum.

While neighboring application and sensing electrodes may be connected, producing a simpler, bipolar configuration requiring only 2 electrodes, the 4-electrode “tetrapolar” method keeps them separate, providing better current density distribution and eliminating the influence of skin-to-electrode contact impedance.

In the presented application, the construction of the anal probe, whose surface contains all the electrodes, is crucial. For study purposes, a commercially available probe (Periprobe RU/AAnalis, manufactured by BeacMed, Italy; 14 mm in diameter, with 2 gold electrodes positioned on opposite sides) was selected and adapted to the needs of impedance measurements. The registrations were performed by setting a pair of electrodes (working in the bipolar configuration) in three different radial orientations and thus obtaining a record from various areas of the sphincter’s circuit:

- along the main axis (longitudinal, front-to-back),
- shifted 60 degrees to the right of the main axis, and
- shifted 60 degrees to the left of the main axis.

**Figure 1:**
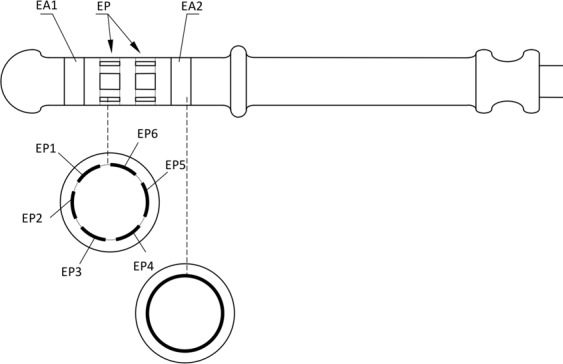
The concept of the target probe with the set of receiving electrodes (EP) and two rings of application electrodes (EA); adapted from patent description^31^.

**Figure 2:**
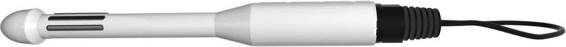
The model of the prototype enabling tetrapolar electrode configuration and comprised with a single set of 4 electrodes.

This resulted in a functional model of a target probe that will work with at least 6 electrodes, forming at least 3 measurement sets in parallel (using the tetrapolar configuration), without the need to rotate the probe inside the anal canal. The concept is presented in Fig. 1. The model of the first prototype allowing the single measurement is shown in Fig. 2.

Bioimpedance data were measured and stored using an Impedimed SFB7 spectrometer (Impedimed, Australia QLD). This is a wireless, battery-powered device, which can be also used outside the office. The single measurement is quick, lasting about 5 seconds after positioning the probe in the anus. The values of resistance and reactance were collected over a frequency range of 3.0–1000.0 kHz. Then, impedance moduli and phase shifts were calculated and used during the analysis.

In order to produce the set of input parameters for comparison with the outputs derived from physical examination, transanal ultrasonography and anorectal manometry, we averaged the values of the moduli and phase shifts (for all radial positions) over 8 (logarithmically equal) frequency subranges: 3.0–6.2 kHz, 6.2–12.8 kHz, 12.8–25.6 kHz, 25.6–54.8 kHz, 54.8–113.2 kHz, 113.2–234.0 kHz, 234.0–483.8 kHz, and 483.8–1000.0 kHz.

Therefore, from each participant, we acquired 48 parameters (impedance modulus and phase-shift curves over 8 frequency subranges for 3 orientations).

### Analysis

We used as output information the following results from the reference techniques (in each case, the two distinguished classes are listed in parentheses):

- rectal physical examination^32^:
  - sphincter tension (correct/reduced)
  - pathologies on the anoderm (present/absent)
  - pathologies in the anal canal (present/absent)
  - pathologies in the rectal mucosa (present/absent)
  - active sphincter contraction (correct/reduced)
  - sphincter continuity (preserved/not preserved)
- transanal ultrasonography^33,34^:
  - up to 50% of the external sphincter thickness torn – 3a of the OASIS classification (yes/no; the participants with more than 50% - 3b in OASIS classification - are also included)
  - more than 50% of the external sphincter thickness torn – 3b of the OASIS classification (yes/no)
  - both external and internal sphincters torn – 3c of the OASIS classification (yes/no)
- anorectal manometry^34^:
  - average resting pressure (correct/reduced)
  - maximal contraction pressure (correct/reduced)
  - duration of effective contraction (correct/shortened)

The values of the diagnostic parameters in the study groups are summarized in Table 1.

**Table 1:** Values of the diagnostic parameters based on physical examination, transanal ultrasonography and manometry in the study groups.

Diagnostic technique	Diagnostic parameter	Result	Number of patients in groups	
			A	B
Physical examination	Sphincter tension	Correct	0	12
		Reduced	10	0
	Pathologies on the anoderm	Absent	10	10
		Present	0	2
	Pathologies in the anal canal	Absent	10	7
		Present	0	5
	Pathologies in the rectal mucosa	Absent	10	6
		Present	0	6
	Active sphincter contraction	Correct	1	11
		Reduced	9	1
	Sphincter continuity	Preserved	10	12
		Not preserved	0	0
Endoanal USG	Up to 50% of the external sphincter torn	Yes	10	0
		No	0	12
	More than 50% of the external sphincter torn	Yes	5	0
		No	5	12
	Presence of internal sphincter torn	Yes	4	0
		No	6	12
Anorectal manometry	Average resting pressure	Correct	2	12
		Reduced	8	0
	Maximal contraction pressure	Correct	1	11
		Reduced	9	1
	Duration of effective contraction	Correct	7	12
		Shortened	3	0

The group was homogeneous in terms of sphincter continuity, and 3 of the 6 parameters (anal, mucosal and anodermal pathologies) from physical examination do not matter in sphincter function evaluation, so we excluded those parameters and used the remaining 8 as the outputs.

**Figure 3:**
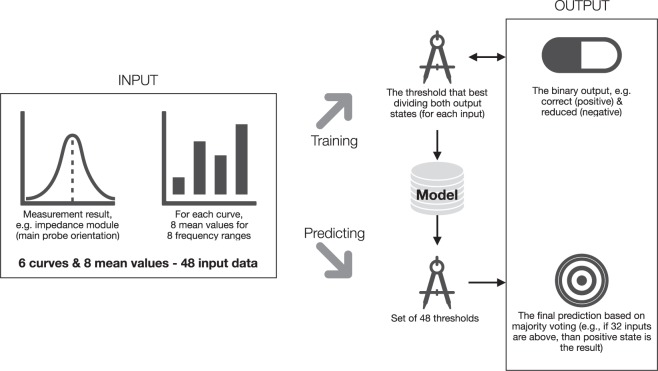
The graphical scheme of the chosen classification procedure for a single parameter, e.g., sphincter tension.

The graphical scheme of the classification analysis is presented in Fig. 3.

We created separate models for each of the reference outputs in the analysis. Each model was designed as a two-state decision box (e.g., correct or reduced) and the final statement was made based on a majority voting process. The set of thresholds was established for all 48 impedance-related parameters (input values). In each case, the threshold was estimated based on the mean of sensitivity and specificity (balanced accuracy metrics, proper in case of class imbalances).

The method efficiency was assessed using 10-fold cross-validation (with a 50%-50% division of data to split even number of patients/controls in both training and testing subsets, respectively). Next, we constructed 2 × 2 confusion matrices. Average sensitivities, specificities, and accuracies were estimated as the ratios of correctly identified positive subjects to all positives, correctly identified negative subjects to all negatives, and correctly identified subjects to all subjects, respectively. Cohen’s kappa coefficient (the measure of inter-rater agreement and accuracy after removing the probable effect of random choice) served as a metric of the comparison between our impedance spectroscopy measurement and a diagnostic value treated as a reference. This coefficient is treated as a clarification metric, an extension of accuracy, which is more robust and considers class imbalances.

All analysis was carried out using software written in R.

## Results

Based on the standard diagnostics tests including physical rectal examination, endoanal ultrasound and manometry, OASIs were found in all 10 women in group A (3a in 5 cases, 3b in 1, 3c in 3 and 4th-degree in 1). In all 6 patients with sphincter defects not detected during delivery, OASIs were confirmed: all perineal tears previously diagnosed as second-degree were found to be third-degree (3a in 3/10, 3b in 1/10 and 3c in 2/10). No sphincter injuries were observed in the control group.

**Figure 4:**
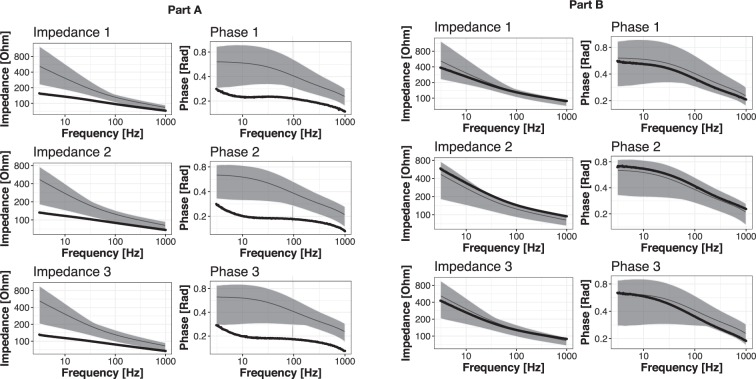
(**A**) (left – injured patient) and (**B**) (right – healthy control). The curves (bold lines) of impedance moduli (1 – main probe orientation; 2 – shifted 60 degrees to the right; 3 – shifted 60 degrees to the left) and phase shifts (the same notations). The range of normative values calculated based on this preliminary study is presented as grey area, with mean value as thin lines.

Figure 4 presents the curves of impedance moduli and phase shifts for the patient (4a) and the control (4b); along with the range of normative values (using grey, based on our preliminary study).

The calculated thresholds (used for classification) of impedance moduli for the orientation along the main axis and within 8 frequency subranges are stored in Table 2 for 8 considered parameters.

**Table 2:** Thresholds of impedance moduli (all in Ohms).

Parameter	3.0–6.2 kHz	6.2–12.8 kHz	12.8–25.6 kHz	25.6–54.8 kHz	54.8–113.2 kHz	113.2–234.0 kHz	234.0–483.8 kHz	483.8–1000.0 kHz
Sphincter tension	317.9	185.1	154.3	153.5	133.8	112.1	98.2	82.6
Active sphincter contraction	317.9	185.1	154.3	118.5	107.2	91.4	98.2	90.5
Up to 50% of the external sphincter torn	317.9	215.1	182.9	153.5	133.8	112.1	98.2	86.6
More than 50% of the external sphincter torn	200.0	215.1	182.9	106.1	92.9	83.9	76.8	70.1
Presence of internal sphincter torn	200.0	155.2	125.8	106.1	92.9	84.2	77.3	70.5
Average resting pressure	207.3	185.1	182.9	106.1	96.8	90.1	77.7	70.8
Maximal contraction pressure	317.9	185.1	154.3	118.5	107.2	91.4	98.2	90.5
Duration of effective contraction	207.3	185.1	161.5	106.1	96.8	90.1	95.2	86.1

The efficiency of the impedance spectroscopy method was quantitatively assessed by sensitivities, specificities, overall accuracies, and Cohen’s kappa coefficients calculated for all the considered outputs from the reference methods. The results are collected in Table 3.

**Table 3:** Summary of impedance spectroscopy accuracy in predicting several parameters established from physical examination, ultrasonography, and manometry.

Parameter	Sens. [%]	Spec. [%]	Accuracy [%]	Cohen’s kappa
Sphincter tension	**70.0**	75.0	72.7	**0.448**
Active sphincter contraction	**70.0**	**80.0**	75.5	**0.499**
Up to 50% of the external sphincter torn	**76.0**	**88.3**	**82.7**	**0.646**
More than 50% of the external sphincter torn	**73.3**	**85.0**	**82.7**	**0.521**
Presence of internal sphincter torn	**70.0**	**90.0**	**86.4**	**0.530**
Average resting pressure	55.0	**80.0**	70.9	0.349
Maximal contraction pressure	**72.0**	**80.0**	76.4	**0.520**
Duration of effective contraction	**70.0**	**84.7**	**82.7**	0.383

Sensitivities of at least 70% were obtained for all considered parameters except average resting pressure, with the highest for the incomplete extent of the external sphincter torn.

Specificities of 80% or above were achieved for all considered parameters from endoanal USG and manometry, as well as for one of the physical examination parameters (active sphincter contraction).

The highest overall accuracies were obtained for the assessment of whether the external and internal sphincters were torn, and for the duration of effective contraction. The impedance parameters attained 74.1% mean accuracy compared to physical examination; 83.9% compared to ultrasonography; and 76.7% for manometry.

In all cases, Cohen’s kappa was greater than 0.3, permitting the statement that impedance data can be used to predict the considered parameters better than random selection. Due to the small amount of data and the class imbalances, the method was primarily treated as a clarification and accuracy extension. Therefore, based on the results, we decided to arbitrarily focus on those parameters for which Cohen’s kappa was greater than 0.4 (treated as a moderate or substantial concordance, especially relevant in assessing human populations):

- sphincter tension,
- active sphincter contraction,
- assessment of whether external sphincter is torn,
- both sphincters torn, and
- maximal contraction pressure.

These allow the inference that impedance data can be used to evaluate parameters from both USG and manometry. They also complement the physical examination. High specificities (in the range of 80–100%) suggest the validity of using the impedance method as a screening test.

## Discussion

The inadequate rate of OASI detection resulting from occult tears and missed diagnoses^11,12^ indicates the need to look for new, non-standard diagnostic tools, easier to use and more widely available in gynecology and surgery units^13,35^.

Impedance seems an objective physical parameter that reflects the features of tissue microstructure (as compared to endoanal ultrasonography) and function disorders (similarly, connected with anorectal manometry). It measures electrical resistivity^19–21^ and its values for proper and damaged structure therefore differ for each tissue^22,23^. The frequency of the current applied during impedance spectroscopy determines the depth of measurement. Any deviations from the regular histological structure will be reflected in the impedance value^24^.

Our study showed that the impedance spectroscopy parameters allow precise assessment of the external anal sphincter, from both anatomical and physiological perspectives. They also allow assessment of internal sphincter parameters with more than 80% accuracy compared to references. If physical rectal examination failed to obtain precise data on sphincter state, impedance testing would allow evaluation of sphincter tension and active sphincter contraction ability with relatively high accuracy. This can be very useful especially after complicated deliveries, when physical examination is difficult and sphincter problems most frequent. The extent to which the problem is overlooked can be seen even in this small study group, where 6 of the 10 cases of OASI were not recognized at delivery.

Furthermore, the parameters obtained from impedance spectroscopy reflect information obtained from endoanal USG and manometry. The former, treated as a gold standard method^36^, is generally accepted as the most sensitive tool for assessing the sphincter complex for the presence of defects or structural alterations. It enables visualization of the anal canal tissues and assessment of the presence or absence of sphincter injuries^37,38^. However, it is specialist test burdened by the need for highly specialized medical staff, not always available in maternity wards^13,35^, and by some technical limitations resulting in patient discomfort especially in the first postpartum period^39^.

Similarly, in the case of manometry, the test has limited availability and is long and uncomfortable. It is also relatively difficult to plan treatment based only on its results, because they do not correlate with clinical manifestation of injury extension. Manometry mainly detects contractions that occlude the lumen, while minor phasic contractions and changes in tone may remain undetected^40^. Its results are usually interpreted along with those of other studies.

In contrast to these standard diagnostic methods, impedance spectroscopy can be used even by inexperienced staff, and thanks to a short, non-invasive measurement with the small-diameter endoanal probe allows use shortly after delivery. In the current setup, the subjects relaxed their muscles, but still, the information about the function is present as functional capabilities are connected with a specific anatomical scheme and physicochemical properties. However, we also consider extending the protocol by imposing to contract the muscles in further studies, which could improve the convergence with manometry results.

This method would help in identifying patients requiring more specialized diagnostics (such as ultrasound and manometry), or even surgical repair of sphincters while still staying in the maternity ward. This would increase the percentage of primary sphincter repairs or delayed primary and early secondary reconstructions (carried out up to 14 days after delivery) which yielded acceptable long-term functional outcomes without the need for a covering stoma^41^.

The obtained metrics, like sensitivities, specificities and accuracies, imply the use of impedance spectroscopy as a screening tool, which gives a chance to significantly reduce the incidence of fecal incontinence in women upon long-term follow-up. To our knowledge, there are no reports in the literature of impedance being used to measure the modality of sphincter injuries; our approach seems to be novel. Based on our preliminary results, we believe that impedance can be utilized as a precise and fast technique for detecting deviations in the anorectal area resulting from sphincter rupture, stretching or other dysfunctions. Since impedance spectroscopy exhibits such a high accuracy, it would answer the needs of perinatal care units to ensure high-quality, professional obstetrical service for women just after childbirth.

We must note the risk of impedance spectroscopy detecting even minor damage which might never become symptomatic. We will address this issue in ongoing studies (on risk management and suitable algorithms) by controlling and possibly negating the bias of results presented to the diagnostician, avoiding misinterpretation in clinical practice.

Considering that the measurements employed a prototype which will be further developed, the method might be applied as a diagnostic tool to accurately assess all the structural elements of the anorectal area. The final cost of a single test for a patient is difficult to predict at this stage, though we presume to deliver a cheap, fast and easily available diagnostic tool.

Presented paper is a continuation to our previous one^42^, which assessed the feasibility to use impedance data to determine classification to issued or control group. The main achievement of this work was the investigation of several specific parameters related to three main techniques (rectal physical examination, transanal ultrasonography, and anorectal manometry) and the efficiency of their classification. This study separates from the previous one as different mathematical approaches and techniques were used.

### Limitations of the study

This was a very small group of subjects, so the presented results should be treated as preliminary. In many cases, the considered classes were highly imbalanced, preventing proper learning of both states during training. Due to these weaknesses, we used very simple classification, consisting of thresholding and majority voting.

Because the study was performed by a single investigator and only once for each patient, an intra/interobserver reproducibility analysis was not performed. To allow more detailed inference, a multi-center study (enabling assessment of inter-observer variability and adding a control group with recent vaginal delivery without any injury) on a larger population is planned.

## Summary

Perianal impedance spectroscopy is a novel, non-invasive technique with potential for detecting obstetrical anal sphincter injuries. Complementing the physical examination, it gives a chance to assess the state of the anal sphincters, primarily before one is able to perform highly specialized diagnostics requiring high capacity for interpretation. It seems to be an appropriate tool to measure and quantify the extent of anal sphincter injury just after vaginal delivery.

## Data Availability

The authors undertake to provide all materials, data and associated protocols promptly to the reader’s request without undue qualifications in material transfer agreements.
